# Post-operative perfusion and diffusion MR imaging and tumor progression in high-grade gliomas

**DOI:** 10.1371/journal.pone.0213905

**Published:** 2019-03-18

**Authors:** Matthew L. White, Yan Zhang, Fang Yu, Nicole Shonka, Michele R. Aizenberg, Pavani Adapa, Syed A. Jaffar Kazmi

**Affiliations:** 1 Radiology, University of Nebraska Medical Center, Omaha, Nebraska, United States of America; 2 Biostatistics, University of Nebraska Medical Center, Omaha, Nebraska, United States of America; 3 Internal Medicine Division of Oncology & Hematology, University of Nebraska Medical Center, Omaha, Nebraska, United States of America; 4 Neurosurgery, the University of Nebraska Medical Center, Omaha, Nebraska, United States of America; 5 South Texas Radiology Group, San Antonio, Texas, United States of America; 6 Anatomic Pathology, Geisinger Medical Center, Danville, Pennsylvania, United States of America; George Washington University, UNITED STATES

## Abstract

**Purpose:**

Perfusion and diffusion magnetic resonance imaging (MRI) provide important biomarkers for brain tumor analysis. Our aim was to investigate if regions of increased perfusion or tumor with restricted diffusion on the immediate post-operative MRI examination would be predictive of time to tumor progression in patients with high-grade gliomas.

**Materials and methods:**

Twenty-three patients with high-grade gliomas were retrospectively analyzed. We measured the perfusion at the resection area and evaluated the presence or absence of the restricted diffusion in residual tumor masses. The associations of the perfusion, diffusion and contrast enhancement (delayed static enhancement (DSE)) characteristics with time to tumor progression were statistically calculated. We also evaluated if the location of the tumor progression was concordant to the areas of the elevated perfusion, tumor type restricted diffusion and enhancement.

**Results:**

Patients with >200 days to progression are more likely to have no elevated relative cerebral blood volume (rCBV) ratio (p = 0.0004), no tumor restriction (p = 0.024), and no DSE (p = 0.052). The elevated mean rCBV ratio (p<0.001) and tumor type restricted diffusion (p = 0.002) were significantly associated with a higher risk of progression. All cases with rCBV ratio of >1.5 progressed in 275 days or earlier. Tumors tended to progress at the area where patients with post-operative MRIs showed elevated perfusion (p = 0.006), tumor-type restricted diffusion (p = 0.005) and DSE (p = 0.008).

**Conclusions:**

Post-operative analysis of rCBV, tumor type restricted diffusion and enhancement characteristics are predictive of time to progression, risk of progression and where tumor progression is likely to occur.

## Introduction

The post-operative MR imaging examination following high-grade glioma surgery has demonstrated importance. The contrast enhancement (delayed static enhancement (DSE)) characteristics are noted around surgical cavity [1–5]. The importance for evaluating the extent of tumor resection as demonstrated by DSE has recently been reconfirmed [2–4]. However, surgery induced DSE can have a similar appearance to tumor DSE. Early post-operative magnetic resonance imaging (MRI) after surgery decreases the amount of post-operative induced DSE but does not eliminate the problem [6].

Dynamic MRI contrast enhancement, e.g. tumor perfusion, is an important biomarker of high-grade gliomas (HGGs) [7]. However, there has been very limited study of immediate post-operative MRI tumor perfusion characteristics. Perfusion weighted MRI (PWMRI) may benefit the immediate post-operative brain tumor MRI exam. Areas of abnormal perfusion in brain tumors overlap but differ compared to the areas of DSE [8, 9]. This suggests PWMRI may help detect high-grade tumor in areas lacking DSE. Furthermore, PWMRI could potentially help discriminate post-surgical DSE from tumor related DSE by demonstrating increased perfusion in areas of true residual tumor.

Restricted diffusion abnormalities in gliomas have been found in higher-grade tumors and can predict survival [10–13]. Low apparent diffusion coefficient (ADC) measurements correlate to hypercellularity in high-grade gliomas [13, 14]. Residual areas of tumor after surgery with hypercellularity should indicate poor prognosis and might indicate where tumor progression should occur.

Evaluating tumor perfusion and diffusion characteristics on the post-operative brain tumor MRI exam should provide a better analysis of the proven importance of the true extent of surgical resection. We hypothesized in HGGs that the post-operative MRI exams with regions of increased perfusion or tumor with restricted diffusion would be predictive of time to tumor progression and of where tumor progression would occur.

## Materials and methods

### Patients

We retrospectively reviewed the medical records and MR images of 23 consecutive patients (13 males and 10 females; age range, 29.3–81.8 years; mean age, 52.4 years) with pathologically proven HGGs. All patients underwent post-operative MR imaging within 24 hours following tumor resection, including dynamic susceptibility contrast PWMRI. Gross total tumor resection and subtotal tumor resection were achieved in 14 and 9 patients, respectively. The pathologic diagnosis was determined with specimens removed at surgical resection by a board-certified neuropathologist utilizing the 2007 World Health Organization Classification of Tumors of the Central Nervous System [15]. Sixteen gliomas were glioblastomas (GBM, WHO grade IV), 5 were anaplastic astrocytoma (AA, grade III), 1 was anaplastic oligodendroglioma (AO, grade III), and 1 was high-grade astroblastoma (Table 1). Cases were not included in this study when susceptibility artifacts from surgical clips, air and blood products obscured the post-operative cavity on the MRI. We also excluded patients without sufficient post-operative follow up.

**Table 1:** Post-operative MR imaging and tumor progression in high-grade gliomas.

							tumor progression				
patient	sex/age	Glioma	tumor resection	rCBV ratio in resection area	resudial tumor-type restricted diffusion	Residual Tumor Enhancement	Concordant to elevated Perfusion area	Concordant to Restricted Diffusion area	Concordant to Enhancement area	treatment	days to recurrence
1	F/81.8	GB	subtotal	2.72	Yes	Yes	Yes	Yes	Yes	CTX+XRT	60
2	F/65.1	GB	total	3.44	No	Yes	Yes	N/A	No	CTX+XRT	186
3	M/34.9	GB	subtotal	2.53	No	Yes	Yes	N/A	Yes	CTX+XRT	124
4	M/52.6	GB	subtotal	2.01	Yes	Yes	Yes	Yes	Yes	CTX+XRT	94
5	F/62.0	GB	total	2.49	Yes	Yes	Yes	Yes	No	CTX+XRT	104
6	M/63.7	GB	subtotal	1.60	No	Yes	Yes	N/A	Yes	CTX+XRT	138
7	M/64.5	GB	total	1.00	No	Yes	N/A	N/A	No	CTX+XRT	264
8	M/43.4	GB	total	1.00	No	Yes	N/A	N/A	No	CTX+XRT	298
9	F/51.9	GB	total	1.76	No	No	Yes	N/A	N/A	CTX+XRT	275
10	M/35.8	GB	total	2.75	Yes	Yes	Yes	Yes	Yes	CTX+XRT	97
11	M/76.5	GB	total	2.91	No	Yes	Yes	N/A	Yes	CTX+XRT	97
12	F/65.9	GB	subtotal	3.89	No	Yes	N/A	N/A	Yes	CTX+XRT	100
13	F/62.4	GB	total	1.00	No	Yes	N/A	N/A	No	CTX+XRT	616*
14	M/48.2	GB	total	1.00	No	Yes	N/A	N/A	No	CTX+XRT	955
15	M/46.9	GB	subtotal	2.64	No	Yes	Yes	N/A	No	CTX+XRT	245
16	M/38.2	GB	subtotal	2.28	No	Yes	Yes	N/A	Yes	CTX+XRT	94
17	M/46.0	AA	total	2.46	No	Yes	N/A	N/A	No	CTX+XRT	217
18	M/32.5	AA	total	1.00	No	No	N/A	N/A	N/A	CTX+XRT	698
19	F/41.8	AA	total	1.34	No	No	N/A	N/A	N/A	CTX	958*
20	F/29.3	AA	total	1.00	No	Yes	N/A	N/A	No	XRT	847*
21	F/63.6	AA	subtotal	1.00	No	No	N/A	N/A	N/A	XRT	460
22	F/54.5	AO	subtotal	1.25	No	Yes	N/A	N/A	No	CTX	832*
23	M/43.1	AB	total	1.00	No	No	N/A	N/A	N/A	XRT	795*

Note: AA, anaplastic astrocytoma; AO, anaplastic oligodendroglioma; GB, glioblastoma; AB, astroblastoma; CTX, chemotherapy; XRT, radiation.

*no recurrence on days when we evaluated patients in this study.

Following the surgical resection, 18 patients received concurrent chemoradiation therapy with temozolomide, 3 patients had chemotherapy alone, and 2 patients underwent radiation alone (Table 1). The patients underwent serial MRIs for the evaluation of tumor stability after postoperative treatment. Response Assessment in Neuro-Oncology Criteria (RANO criteria) were used for assessing the tumor progression based on clinical symptoms and imaging features on follow up MRIs [16]. Follow up was obtained per NCCN guidelines. GBMs were followed by MRI every 2 months and then every 3 months if patients were off therapy for more than a year. AAs and the AO were followed up by MRI every 2–3 months. Post-operative ischemia was detected with diffusion weighted imaging (DWI) and utilized to identify areas of ischemia induced DSE on follow-up imaging. The time of progression was retrospectively corrected to the time when the changes first appeared in the region that were eventually proven to be progression [16]. We defined a time threshold to differentiate the patients into 2 groups, those with progression before 200 days and those after 200 days from the immediate post-operative MR examination.

The study was approved by the Institutional Review Board at University of Nebraska Medical Center. Informed patient consent was not required for the retrospective review of the medical records or the MR images for this study.

### MRI technique

The post-operative MRI examinations are summarized as follows: 11 patients on Achieva (3T, Philips Medical Systems, Best, The Netherlands), 7 patients on Intera (1.5T, Philips Medical Systems, Best, The Netherlands), 3 patients on Signa HDx (3T, GE Healthcare, Milwaukee, WI), and 2 patients on Signa HDxt (1.5T, GE Healthcare, Milwaukee, WI). On all MR systems, dynamic susceptibility-weighted perfusion contrast-enhanced MR images were acquired during the first pass of a standard-dose (0.1 mmol/ kg) bolus at a rate of 5 mL/s. The contrast used was gadobenate dimeglumine (n = 22, MultiHance Bracco, Milan, Italy) or gadobutrol (n = 1, Gadavist, Schering, Berlin, Germany). The conventional MR study included the following sequences: T1, T2, FLAIR, DTI (using b-values of 800 on Philips and 1000 on GE), and postgadolinium axial, coronal and/or sagittal T1 sequence. Twenty patients underwent contrast DSE high spatial resolution 3D T1-weighted imaging, and transverse images were reformatted from that data set.

Perfusion imaging was conducted on the Philips MR scanners using the following parameters: TR/TE, 15ms/23ms; field of view, 220mm x 220mm; slice thickness, 3.5mm; slice gap, 3.5mm; NEX, 1; matrix, 128 x 128 x 16; flip angle, 7. This is PRESTO (principle of echo shifting with a train of observations; Philips Medical Systems, Best, The Netherlands) technique.

The perfusion parameters on the GE MR scanners were as follows: TR/TE, 2200ms/20ms on 3T and 1900ms/80ms on 1.5T; field of view, 260mm x 260mm on 3T and 300mm x 300mm on 1.5T; slice thickness, 5mm; slice gap, 5mm; NEX, 1; matrix, 128 x 128 x 16; flip angle, 60 on 3T and 90 on 1.5T.

### MR imaging analysis

MR images were transferred to a personal Linux workstation and processed with a series of imaging software packages, including FMRIB's Software Library (FSL) v5.0 and ImageJ, and in-house built tools. In each case, all imaging modalities were rigidly registered to the T1 post-contrast image using the brain extractions (FSL's BET tool). Perfusion data was analyzed with the ImageJ package (v1.42) producing rCBV maps corrected for contrast leakage with the DSCoMAN plugin applied to the motion-corrected (FSL's mcflirt) image set [17].

A board certified neuroradiologist (M.L.W) and a radiologist (Y.Z) analyzed the images and reached a consensus regarding the extent of resection, location of the residual tumor and the presence of postsurgical injury. Both radiologists were blinded to the grade of tumors, MR reports and the surgical result (total or subtotal resection) when performing imaging analysis. The elevated perfusion visually found in the resection area on the rCBV map was the Region of interest (ROI) for measuring the rCBV. The rCBV ratio was calculated by comparing the rCBV with a measurement of the contralateral normal white matter, i.e., the ipsilateral value was divided by the contralateral value. If no elevated perfusion was noted then an rCBV ratio of 1.0 was used indicating perfusion was the same as normal brain. The volumes of the ROIs were recorded.

The presence or absence of the residual DSE and restricted diffusion were evaluated. Post-operative DSE was evaluated as thick and/or nodular consistent with tumor, thin rim enhancement and no enhancement. Restricted diffusion was analyzed as having an appearance of surgical induced ischemic change versus restricted diffusion in residual tumor mass (tumor-type restricted diffusion). Surgical induced ischemic change is often a thin rim of restricted diffusion at the post-surgical cavity or restricted diffusion in a vascular distribution related to the surgical site. We inspected pre-operative MRI to determine restricted diffusion related to tumor or surgery. We also confirmed the location of the tumor progression on follow up MR images to see if the progression is concordant to the area of the elevated perfusion, DSE (tumor type or thin rim), or tumor-type restricted diffusion on the initial post-operative MR exam.

### Statistical analysis

Data analysis was performed by a biostatistician (F.Y). The primary outcome variable is time to tumor progression. The goal of the analysis is to identify important demographical, clinical or imaging variables that are associated with time to tumor progression. The demographical and clinical variables included the patient age, sex, and treatments received. First, the Wilcoxon rank test and Fisher exact test were used separately to compare the continuous or categorical variables between the two groups of patients with different length of time to progression (i.e., before 200 days or after 200 days since the initial post-operative MR examination). Then, Cox proportional hazard regression was used to evaluate the association between each demographical or clinical variable with time to progression separately. In addition, a sensitivity analysis restricted to the subjects treated with both chemotherapy and radiation was conducted to evaluate the association of those variables with time to progression to remove the influence of the treatment effects in the analysis. IDH mutation data were not available for most patients in this study. Given this we could not evaluate the effect of IDH mutation on tumor progression.

## Results

**Fig 1:**
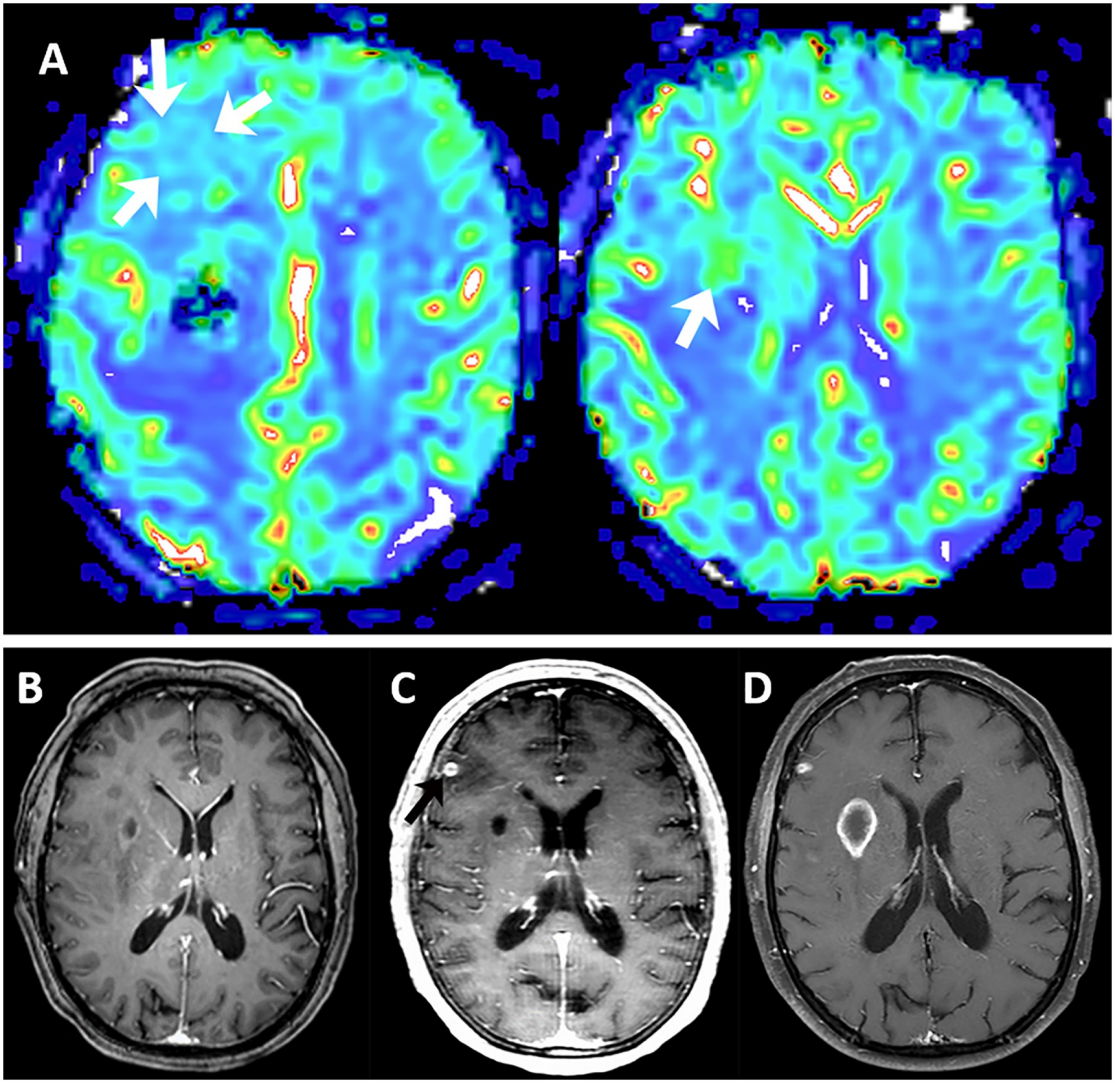
Patient with GBM and a near total resection. A). rCBV maps demonstrate focuses of elevated perfusion diffusely in the right frontal lobe and in the right basal ganglia (arrows). B) post-op T1 image with gadolinium demonstrates a cystic lesion with minimal enhancement in the right basal ganglia. C) 100 days post-operative MRI demonstrated tumor progression with development of a small nodule of cortical enhancement in the right frontal lobe (arrow). D) The next follow-up MRI demonstrates progression of the abnormalities with development of enhancement associated with the basal ganglia cystic lesion.

**Fig 2:**
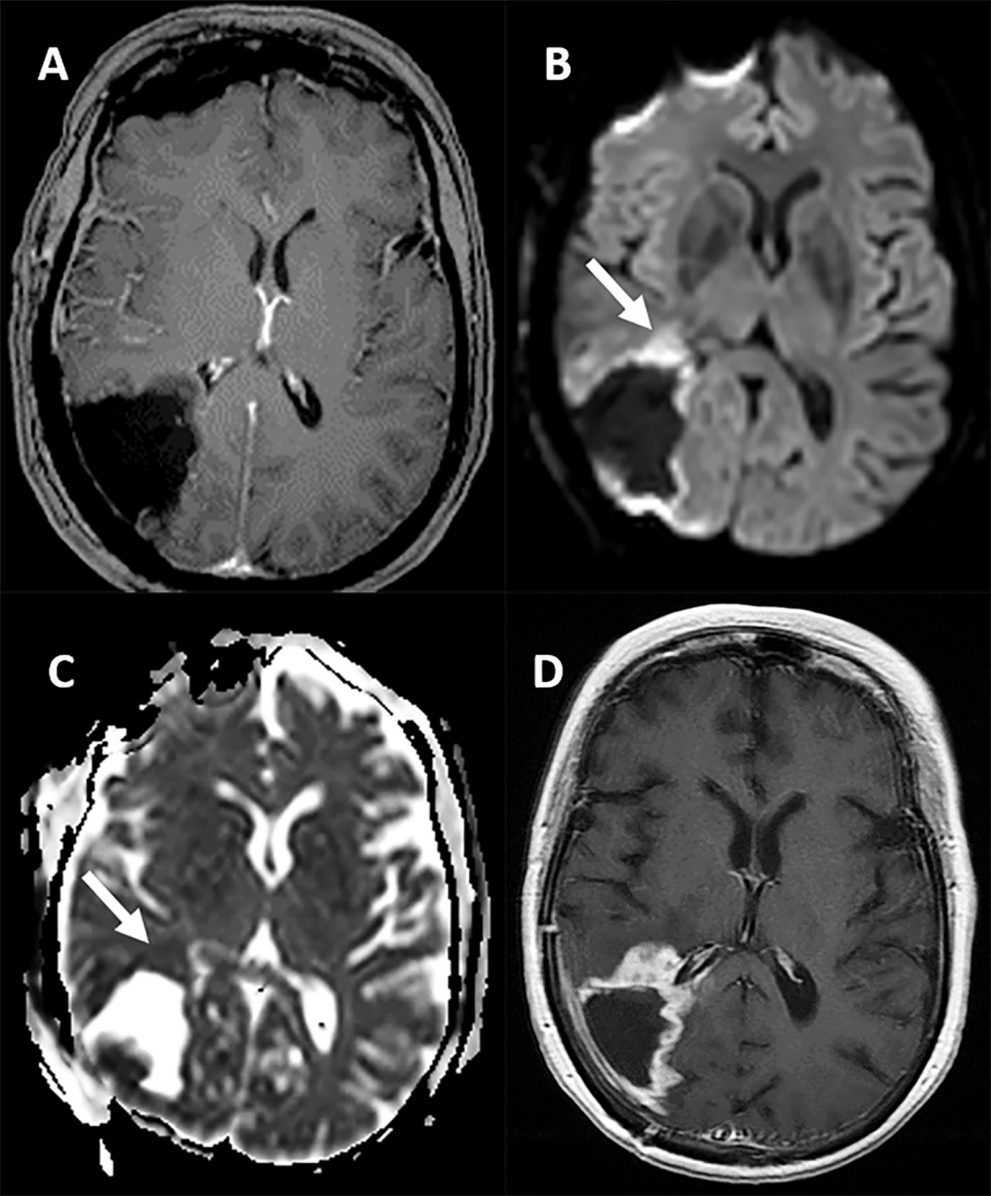
Patient with GBM and a total tumor resection. Patient with GBM and a total tumor resection. A). post-op T1 image with gadolinium demonstrates a cystic cavity without enhancement in the right parietal lobe. There is a rim of restricted diffusion at the margin of the cavity on B) DWI and C) ADC map, representing post-operative change. Note the nodular-like area with restricted diffusion (arrows) anterior to the cavity, which developed a nodular area of tumor progression on the 105 days post-operative MRI (D).

**Fig 3:**
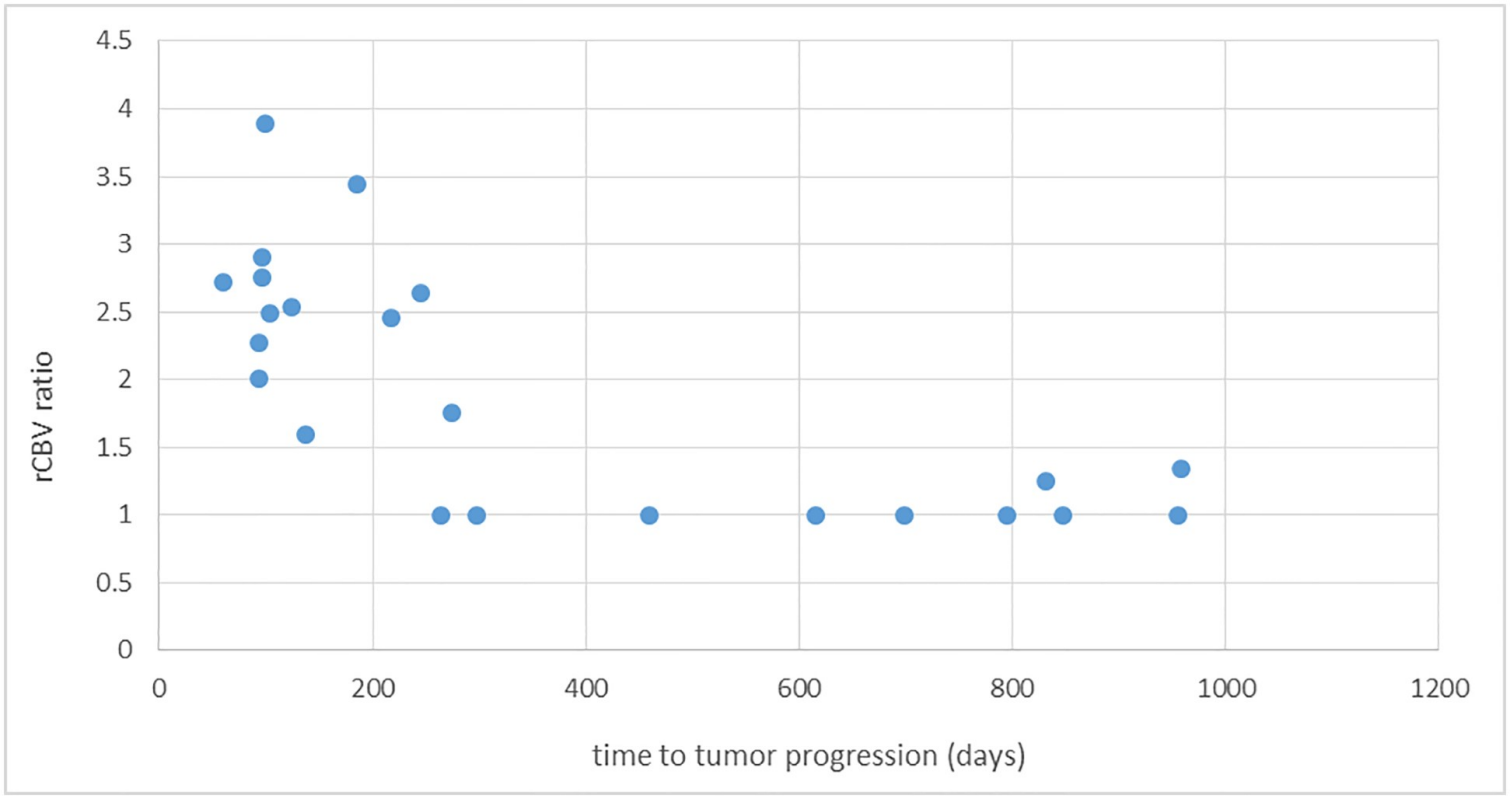
Time to tumor progression vs rCBV ratio in high-grade gliomas. The graph demonstrates patients with a longer time to progression are more likely to not have tissue with an elevated rCBV ratio post-operatively. All cases with rCBV ratio of >1.5 progressed in 275 days or earlier.

Of the 23 patients, 15 patients had elevated perfusion in the resection area on the rCBV maps. The mean and standard deviation of the rCBV ratio in these 15 patients was 2.40 ± 0.73. In the remaining 8 patients, the rCBV ratio of 1.0 was used indicating perfusion in the resection area was the same as normal brain. The presence of residual tumor-type restricted diffusion and DSE were found in 4 and 18 patients, respectively. Nineteen patients had surgical induced restricted diffusion. The location of the tumor progression was concordant to the area of the elevated perfusion in 11 out of 15 (73%) patients, to the area of the tumor-type restricted diffusion in 4 out of 4 (100%), and to the area of DSE in 9 out of 18 (50%). There were 8 cases that had no evidence of enhancing tumor post-operatively but had elevated perfusion (rCBV 1.4–3.9). The recurrent tumor in six of these cases occurred at areas of residual post-operative high perfusion. All cases with rCBV ratio of >1.5 progressed in 275 days or earlier (Figs 1, 2 and 3) (Table 1).

The mean rCBV ratio in the 13 patients with 200 days or more for progression was 1.34 ± 0.56. The mean rCBV ratio in the 10 patients with progression within 200 days was 2.66 ± 0.63. The former was significantly lower than the latter (p = 0.0004). Also, patients with <200 days to progression are more likely to have tumor-type restricted diffusion (p = 0.024) and DSE (p = 0.052). In these patients there was concordance in elevated perfusion (p = 0.0015) or DSE (p = 0.015) with the areas of tumor progression. Other variables (age, sex, and volumes of the elevated perfusion) showed no significant differences between these two groups with less than vs more than 200 days for progression (p>0.05).

The mean rCBV ratio (p<0.001) and tumor-type restricted diffusion (p = 0.002) by the Cox proportional hazard regression model were significantly associated with higher risk of progression. For example, the relative risk of having tumor progression association with 1 unit increase in mean rCBV is 3.45 (95% CI = 1.80 to 6.63). Additionally, the relative risk for the patients with tumor-type restricted diffusion vs others equals 11.92 (95% CI = 2.57 to 55.23). Cox proportional hazard found significance associated with the presence of recurrent tumor being concordant with areas of elevated perfusion (p = 0.006), tumor-type restricted diffusion (p = 0.005) and presence DSE (p = 0.008).

## Discussion

The post-operative MRI examination after brain tumor surgery is a critical exam that is necessary to evaluate the extent of surgical resection of a brain tumor and to evaluate for post-operative complications. The importance for evaluating the extent of tumor removal by DSE has been well demonstrated [2–6]. To further characterize the post-operative MRI examination and what it can predict about tumor progression, we evaluated the post-operative perfusion and diffusion characteristics in HGG brain tumor patients. Given previous published findings we also evaluated standard DSE. Additionally, we specifically evaluated where these post-operative abnormalities were in relationship to where tumors progressed using RANO criteria. This type of analysis is potentially very important. If it is determined that where an abnormality is present is where tumor progression occurs, then potentially this is more indicative of where a successful intervention might be aimed.

We found time to tumor progression to have a strong relationship to presumptive residual areas of tumor with elevated rCBV on the immediate post-operative MRI examination. The finding of tumor-type restricted diffusion and DSE characteristics at the operative site were also indicators of a shorter time to or higher risk of progression.

Our findings support our hypothesis that elevated immediate post-operative residual CBV abnormality at the operative site is predictive of early tumor progression. This is likely based on the relationship of CBV and angiogenesis [18, 19]. There was also concordance of where the elevated rCBV was located to where tumor progression subsequently occurred. The elevated rCBV values in tumors presumptively reflect underlying angiogenesis, which is a factor that drives primary brain tumor behavior, and elevated CBV in primary brain tumors predicts increased microvasculature [18–20]. Essock-Burns et al. showed abnormal microvasculature existed beyond the DSE and was significantly reflected by increased CBV [19].

The basis for analyzing tumor perfusion, rather than just DSE tumor, is these techniques evaluate different physiological phenomena and are not always associated with each other [8, 9, 21]. There is very limited information on perfusion characteristics of HGGs on the immediate post-operative MRI examination. Lee EK et al. did study perfusion on the immediate post-operative exam in subjects with glioblastoma [22]. They found the perfusion abnormality present was predictive of one-year progression [22]. This work is supportive of our findings but was in a different population (only GBM) utilizing a different analytic approach. Very importantly, Lee EK et al. did not evaluate for perfusion abnormalities in cases of gross total resection determined by contrast enhancement (DSE). This approach limits the utility of post-operative perfusion analysis since elevated perfusion can exist in gliomas when there is no contrast enhancement. For instance, eight of our cases had gross total resection and no tumor enhancement post-operatively but had residual elevated perfusion. Six of these cases had progression concordant to where the high perfusion was located. In addition, Lee et al did not analyze the correlation of where tumor progressed and where the perfusion abnormalities were detected [22]. In addition, our methodology in cases of HGG allows prediction of who will progress sooner than at 1 year. In fact, all cases with residual rCBV >1.5 progressed at 275 days or earlier and there were statistically significant differences for perfusion, diffusion and DSE for patients that progressed before and after 200 days.

Other authors have found that the additional information that PWMRI provides can be useful for predicting progressive disease, but the PWMRI exams analyzed in these papers were not performed post-operatively (they were performed pre-operatively), and the results of the PWMRI analysis were mixed [8, 9, 21]. The technique of analyzing tumor progression and growth post-operatively by biomarkers on pre-operative perfusion studies is a potential methodological limitation and does not address adequately what component of tumor with elevated CBV is resected.

Analysis of DWI was made since the literature has demonstrated that areas of restricted diffusion in primary brain tumors are predictive of survival, progression and are a bad prognostic indicator in gliomas. We found that when tumor-type restricted diffusion was present post-operatively, not due to surgical induced ischemia, this correlated with a higher risk of progression. However, this was present in only 4 subjects. The finding of post-operative tumor-type restricted diffusion being a prognostic indicator of progression is supported by the literature, where intrinsic diffusion abnormalities in gliomas have been found to represent higher grade tumor and predict survival [10, 11, 13].

The contrast DSE characteristics of the postoperative cavity have been extensively studied [1–5]. We found the presence of no DSE at the operative site was predictive of a longer time to tumor progression (>200 days). Also, there was a significant association of concordance where the postoperative DSE was present and where the tumor recurrence occurred.

We analyzed where the post-operative abnormalities were located in relationship to where tumor progression occurred. Tumors tended to progress in concordance with location on post-operative MRIs where there was elevated perfusion (p = 0.006), and tumor-type restricted diffusion (p = 0.005) and DSE (p = 0.008). When these three factors were detected, on the post-operative MRI exam, progression was likely to occur. This is expected since these findings are indicative of the presence of tumor.

There were limitations to this study. The number of patients was small because we only enrolled patients with perfusion imaging within 24 hours following tumor resection and also with sufficient post-operative follow-up. Also, patients were excluded in this study when the visible image artifact from surgical clips, air and blood products were present. Measuring residual perfusion post-operatively can be confounded by potentially measuring normal cortex which is distorted instead of only measuring residual tumor. The perfusion analysis is complicated by the potential of gadolinium leakage secondary to breakdown of the blood brain barrier [17]. We did employ the Boxerman algorithm in order to decrease the effects of this on our data [17]. Exams were performed on different MRI scanners given the retrospective nature of this work and how clinical cases are scheduled. However, variability resulting from different techniques on the scanners is decreased by utilizing rCBV ratios of the resection areas (ROI of the resection area divided by contralateral normal white matter).

The analysis of DWI is complicated by post-surgical induced restricted diffusion (observed in n = 19). Also, offsetting the ability of ADC analysis to detect restricted diffusion due to hypercellularity is the presence of vasogenic edema, necrosis and hemorrhage within tumors that can alter ADC values. We utilized RANO criteria to define tumor progression without pathological confirmation on all of our cases. However, RANO criteria is the clinical standard that has been developed to define progression and re-operation is not standardly performed in the clinical setting to confirm progression of HGGs. Our pathological analysis utilized the 2007 World Health Organization Classification of Tumors of the Central Nervous System and future analysis with the 2016 World Health Organization Classification of Tumors of the Central Nervous System potentially could offer additional insights to how post-operative imaging characteristics influence HGGs. The comparison literature, which is very limited, also did not utilize the 2016 World Health Organization Classification of Tumors of the Central Nervous System.

The extent of tumor resection (total or subtotal resection) may have a large impact on post-operative tumor progression. In order to more accurately analyze the relationship between image features (rCBV, diffusion, and tumor enhancement) and tumor progression, cases with total or subtotal tumor resection should be analyzed separately. Therefore, further study with more cases is necessary to acquire statistic conclusion.

## Conclusion

Elevated perfusion, tumor type restricted diffusion and DSE characteristics are more likely to be associated with early tumor progression and also be concordant to the location of the tumor progression. However, diffusion and DSE analyses are more difficult since surgery itself can cause restricted diffusion and DSE at the tumor resection site. Post-operative analysis should include PWMRI, DWI and DSE evaluation. Evaluating tumor perfusion and restricted diffusion post-operatively should give a better idea of the true extent of tumor resection than utilizing only DSE. How residual high rCBV tissue or tumor with restricted diffusion should be treated post-operatively needs to be investigated further. Our data indicates tissues with these characteristics are where tumor progression occurs. This information could potentially be utilized to re-operate in order to achieve gross tumor resections or to optimize the radiation therapy plan.

## Supporting information

S1 Table All rCBV measurements from the elevated perfusion found in the resection area. (XLSX)
